# Supplementary material for: HIVprotI: an integrated web based platform for prediction and design of HIV proteins inhibitors
Source: J Cheminform. 2018 Mar 9;10:12. doi: 10.1186/s13321-018-0266-y (PMC5845081; doi:10.1186/s13321-018-0266-y)
Supplement: Supplementary file 2 — Additional file 2. Source code of HIVProtI web server. [file 13321_2018_266_MOESM2_ESM.zip › HIVprotI_Source-code/hivprotI/weka-3-6-6/doc/weka/classifiers/trees/adtree/PredictionNode.html]

PredictionNode


---


|  |  |  |
| --- | --- | --- |
| **Overview** \| **Package** \| **Class** \| **Tree** \| **Deprecated** \| **Index** \| **Help** \| **Weka's home** | |  |
| PREV CLASS   **NEXT CLASS** | **FRAMES**    **NO FRAMES**     **All Classes** |  |
| SUMMARY: NESTED \| FIELD \| CONSTR \| METHOD | DETAIL: FIELD \| CONSTR \| METHOD |  |


---


## weka.classifiers.trees.adtree Class PredictionNode

```
java.lang.Object
  weka.classifiers.trees.adtree.PredictionNode
```

**All Implemented Interfaces:**: java.io.Serializable, java.lang.Cloneable, RevisionHandler

---

``` public final class PredictionNode extends java.lang.Object implements java.io.Serializable, java.lang.Cloneable, RevisionHandler ```

Class representing a prediction node in an alternating tree.

**Version:**
:   $Revision: 1.7 $

**Author:**
:   Richard Kirkby (rkirkby@cs.waikato.ac.nz)

**See Also:**: Serialized Form

---

**Constructor Summary**

`PredictionNode(double newValue)`
  
          Creates a new prediction node.


| **Method Summary** | |
| --- | --- |
| `void` | `addChild(Splitter newChild, ADTree addingTo)`             Adds a child to this node. |
| `java.util.Enumeration` | `children()`             Enumerates the children of this node. |
| `java.lang.Object` | `clone()`             Clones this node. |
| `FastVector` | `getChildren()`             Gets the children of this node. |
| `java.lang.String` | `getRevision()`             Returns the revision string. |
| `double` | `getValue()`             Gets the prediction value of the node. |
| `void` | `merge(PredictionNode merger, ADTree mergingTo)`             Merges this node with another. |
| `void` | `setValue(double newValue)`             Sets the prediction value of the node. |

**Methods inherited from class java.lang.Object**

`equals, getClass, hashCode, notify, notifyAll, toString, wait, wait, wait`

**Constructor Detail**

### PredictionNode

```
public PredictionNode(double newValue)
```

:   Creates a new prediction node.

    **Parameters:**: `newValue` - the value that the node should store


**Method Detail**

### setValue

```
public final void setValue(double newValue)
```

:   Sets the prediction value of the node.

    :   **Parameters:**: `newValue` - the value that the node should store

---


### getValue

```
public final double getValue()
```

:   Gets the prediction value of the node.

    :   **Returns:**: the value stored in the node

---


### getChildren

```
public final FastVector getChildren()
```

:   Gets the children of this node.

    :   **Returns:**: a FastVector containing child Splitter object references

---


### children

```
public final java.util.Enumeration children()
```

:   Enumerates the children of this node.

    :   **Returns:**: an enumeration of child Splitter object references

---


### addChild

```
public final void addChild(Splitter newChild,
                           ADTree addingTo)
```

:   Adds a child to this node. If possible will merge, and will perform a deep copy
    of the child tree.

    :   **Parameters:**: `newChild` - the new child to add (will be cloned): `addingTo` - the tree that this node belongs to

---


### clone

```
public final java.lang.Object clone()
```

:   Clones this node. Performs a deep copy, recursing through the tree.

    :   **Overrides:**: `clone` in class `java.lang.Object`
    :   **Returns:**: a clone

---


### merge

```
public final void merge(PredictionNode merger,
                        ADTree mergingTo)
```

:   Merges this node with another.

    :   **Parameters:**: `merger` - the node that is merging with this node - will not be affected, will instead be cloned: `mergingTo` - the tree that this node belongs to

---


### getRevision

```
public java.lang.String getRevision()
```

:   Returns the revision string.

    :   **Specified by:**: `getRevision` in interface `RevisionHandler`
    :   **Returns:**: the revision


---


|  |  |  |
| --- | --- | --- |
| **Overview** \| **Package** \| **Class** \| **Tree** \| **Deprecated** \| **Index** \| **Help** \| **Weka's home** | |  |
| PREV CLASS   **NEXT CLASS** | **FRAMES**    **NO FRAMES**     **All Classes** |  |
| SUMMARY: NESTED \| FIELD \| CONSTR \| METHOD | DETAIL: FIELD \| CONSTR \| METHOD |  |


---
